# N : P Stoichiometry in a Forested Runoff during Storm Events: Comparisons with Regions and Vegetation Types

**DOI:** 10.1100/2012/257392

**Published:** 2012-04-01

**Authors:** Lanlan Guo, Yi Chen, Zhao Zhang, Takehiko Fukushima

**Affiliations:** ^1^State Key Laboratory of Earth Surface Processes and Resource Ecology, Academy of Disaster Reduction and Emergency Management, Beijing Normal University, Beijing 100875, China; ^2^Key Laboratory of Environmental Change and Natural Disaster, MOE, Beijing Normal University, Beijing 100875, China; ^3^Graduate School of Life and Environmental Science, University of Tsukuba, Tsukuba 305-8572, Japan

## Abstract

Nitrogen and phosphorus are considered the most important limiting elements in terrestrial and aquatic ecosystems. however, very few studies have focused on which is from forested streams, a bridge between these two systems. To fill this gap, we examined the concentrations of dissolved N and P in storm waters from forested watersheds of five regions in Japan, to characterize nutrient limitation and its potential controlling factors. First, dissolved N and P concentrations and the N : P ratio on forested streams were higher during storm events relative to baseflow conditions. Second, significantly higher dissolved inorganic N concentrations were found in storm waters from evergreen coniferous forest streams than those from deciduous broadleaf forest streams in Aichi, Kochi, Mie, Nagano, and with the exception of Tokyo. Finally, almost all the N : P ratios in the storm water were generally higher than 34, implying that the storm water should be P-limited, especially for Tokyo.

## 1. Introduction

Nitrogen (N) and phosphorus (P) are considered the most important limiting elements for vegetation in terrestrial ecosystems, especially for the forested headwater watersheds, where there is no direct application of fertilizer and the soils are commonly considered to be infertile [1, 2]. At longer time scales of ecosystem development, the predominant source of P is from rock weathering while N is of atmospheric origin. Accordingly, plants growing on young soils tend to be N-limited while vegetation on older, highly weathered soils is often P-limited [3–5]. Similarly, P is commonly believed to be the limiting nutrient in the freshwater whereas N as a limiting factor in estuarine or marine waters [6–8]. These broad-scale trends in the aquatic environment, soil features, and climatic conditions, coupled with smaller-scale heterogeneity in environment-vegetation interactions, produce a spectrum of nutrient availabilities and patterns of nutrient limitation [8–13]. As for nutrient-poor forested ecosystems, many researchers have suggested that N alone or N and P together may be limited elements based on the investigations such as decomposition, nutrient mineralization, trace gas emissions, and leaching losses [13–15]. However, most observations have only focused on the foliage, root dynamics, litter, ground vegetation, and the soil, even only a few have been on stream water with the exception of studies on forested streams [15–17].

The N : P ratio has gained worldwide acceptance as an indicator of biological growth and nutrient cycling and has been successfully used in several studies of both aquatic and terrestrial areas [9, 10, 17]. As an important part of forest ecosystems, stream water serves an export function, and creates a bridge between terrestrial and aquatic ecosystems. Accordingly, the N : P ratio in stream runoff from forested watersheds has been used as an index to diagnose the nutrient status of both terrestrial forests and the water body downstream [2, 18, 19]. As the interface between terrestrial and aquatic systems, runoff from a forested watershed is a critical, however, complex process, since the two types of ecosystem have different dynamics which interact together [18, 20]. Additionally, viewed as a space-time matrix of possible outcomes, researchers have assumed that spatial factors predominate in terrestrial systems, and time factors are more critical in aquatic systems [7, 21]. The interface combines extreme hydrologic events (both varying in magnitude and time) with biogeochemical environments, which makes it critical for elucidating nutrient dynamics in the forested ecosystems [22, 23]. Most previous studies have shown the significant role of storm runoff in nutrient cycling relative to baseflow conditions, which is generally considered as an equilibrium state by many ecologists. However, studies on nutrient dynamics and nutrient limitation during the nonequilibrium situations, for example, extreme storms, have been generally ignored [18, 24]. 

In 2003, Turner et al. [25] found seasonal differences in nutrient limitations by investigating their temporal dynamics in soil and a stream, in which marked P limitation was expected throughout the year, with the likelihood of some communities becoming N-limited during the spring. Moreover, in a previous study on runoff from forested watersheds during baseflow conditions, we found that the N : P ratio showed clear regional characteristics, and a uniformly higher N : P ratio than from an evergreen conifer (EC) than deciduous broadleaf (DB) forest among five Japanese forests [2]. But water chemistry in small forest watersheds is very sensitive to precipitation/discharge events [26, 27], particularly in response to extreme ones such as flooding. Considering the vital role of storm runoff in regulating the nutrient cycles in a forest ecosystem [2], it would be beneficial to characterize the nutrient concentration and N : P ratio in streams during storm events. Therefore, our objectives in this study were the following: (1) to characterize the N : P ratios in storm runoff at both regional and ecosystem (forest) scales; (2) to determine the potential factors controlling their spatial variability; (3) to understand what the potential limiting nutrients in storm runoff waters are from forested watersheds. Our study aimed to provide evidence to fill the knowledge gap between terrestrial and aquatic systems, with a special focus on storm events, a nonequilibrium situation which generally receives little attention. 

## 2. Methods

### 2.1. Study Sites

We chose a set of headwater streams located throughout five regions in Japan (Aichi, Kochi, Mie, Nagano, and Tokyo), which were characterized by different climatic, geological, and topographical conditions, matching as closely as possible the following watershed criteria: (1) the presence of first- or second-order streams, (2) the watersheds were entirely forested, and (3) a lack of obvious lakes or wetlands in the watershed. All of the streams were fast flowing and the streambeds were composed of boulders and large cobbles with little accumulation of fine sediment. Within each region we selected two to six streams, for which the vegetation (including evergreen conifer (EC) plantations, and natural deciduous broadleaf forest (DB)) was representative of the region and shared similar geological environments. The topographic, meteorological, and vegetation details of each sampling site are summarized in supplementary material see Table 1 and Figure 1 in Supplementary Material available online at doi: 10.1100/2012/257392).

The Aichi sites were located in the Aichi Research Forest of The University of Tokyo, east of Inuyama in Aichi Prefecture. The watershed was composed mainly of Neogene sediments and had an undulating topography. The Kochi sites were located in the Tsuzuragawa watershed, which is part of the east tributary of the Shimanto River, southeast of Tashouchou in Kochi Prefecture. The terrain comprised somewhat steep and incised hillsides. Sandstone and pelitic rocks were dominant in these areas. The Mie sites were located in Taikichiou, Mie Prefecture, and were characterized by generally steep slopes. The prevalent rock was gneiss, with a typical brown forest soil cover. The Nagano sites were located in the Terasawayama Education and Research Forest of Shinshu University in Ina, Nagano Prefecture. The Tanazawagawa, a small tributary of the Tenryu River, discharges from this area. As in Mie, steep slopes are a common feature of this area. The watersheds were underlain by granite. The Tokyo sites were located in the Joubanzawa watershed, a tributary of the Arakawa River, and near the headwaters of the Narikigawa in Tokyo. Steep, incised hillsides are also common in this area. The watershed is underlain by sandstone, pelitic rocks, and chert.

### 2.2. Field Sampling and Observations

We performed our sampling regime from June 2004 to July 2005 during 10 storm events (not every storm event occurred in every region, and ensured that samples were taken from both the EC and DB streams during the same event and in the same region). Samples were collected in 500 mL clean polyethylene bottles during rainfall storm events using autosamplers (Teledyne Isco Inc., USA, model 6712) which were activated automatically when the stream water level increased. Discrete samples were usually collected half-hourly, or sometimes hourly or two-hourly during the falling limb of the hydrograph. All samples were shipped by refrigerated express to our laboratory and placed in a cool store until analysis. Next, the samples were filtered through precombusted (at 450°C for 3 hours) and preweighed glass fiber filters (0.45 *μ*m Whatman GF/F, Chicago, USA). The filters were dried at 90°C for 24 hours then reweighed. The filtrates were retained at 1°C for further analysis. A series of analyses were carried out on the filtered subsamples. The following were measured using an Auto-analyzer (Traacs 800, Bran Luebbe Co., New York, USA): NH_4_–N+NO_3_+NO_2_–N, as dissolved inorganic N (DIN), the molybdate-reactive fraction of P (DIP), total dissolved N (DTN: persulfate oxidation method), and the total dissolved P (DTP: persulfate oxidation method). Dissolved organic N (DON) and dissolved organic P (DOP) were determined as the difference between DTN and DIN and that between DTP and DIP, respectively. The N : P ratio was calculated from the molar ratio of DTN and DTP.

### 2.3. Data Analysis

Descriptive statistics were first conducted to investigate the nutrient concentrations for the complete dataset, and to make comparisons with baseflow conditions. Then we investigated the correlation between the water quality indicators. We grouped the data by region, and performed a one-way analysis of variance (ANOVA) to examine the regional distribution of N and P constituents and the N : P ratio. The Turkey multiple comparison and F-test were used to identify significant differences. We also conducted ANOVA analyses to investigate the differences between the two vegetation types, that is, the EC and DB forests and the factors influencing the concentration of N and P and the N : P ratio. Finally, nutrient limitations and during storm flow conditions were discussed.

## 3. Results

### 3.1. Characterization of Water Quality in Storm Runoff

Table 1 showed that higher concentrations of dissolved N, and P occurred in storm flow than during baseflow conditions with the exception of DIP. The means for DTN, DTP, DIN, and DOP during storm flows were about double those during the baseflow conditions. Moreover, the mean DON reached a peak 4.8-times greater than that during baseflow conditions. As for the N : P ratio, the mean in stormflow water flow was about 1.4-times greater than at baseflow. Furthermore, we found that DIN was the dominant N component, being 28.7 and 17.9 *μ*mol L^−1^ higher than DON concentrations during baseflow, and stormflow conditions, respectively. Of the inorganic N, NO_3_–N was the dominant constituent, comprising 55 to 95% in storm runoff for each site (data not shown). However, DOP concentrations were the dominant portion of DTP (accounting for 62–75% of the total) in storm runoff, and the concentrations of DOP were also higher on a weekly basis than during baseflow conditions.

The correlation analysis revealed that there were strong positive correlations between any two parameters, with the exception of the correlation between DON and DOP (*r* = 0.081, *P* = 0.062; Table 2). The significant correlations (all  *P* < 0.001) between both the inorganic components and others indicated a potential similarity in factors controlling the nutrient runoff from forested watersheds during storm events. However, the lack of a correlation between two organic components (DOP and DON) implied the presence of a complex mechanism controlling their runoff during extreme storm events.

### 3.2. Regional Characteristics of Nutrient Concentrations and N : P Ratios during Storm Events

Since many environmental factors (including climate, topography, and soil) interact on a regional scale [28], we summarized factors controlling water quality for each site, and grouped them into five regions (Table 3). There were regional differences in nutrient concentrations among the five regions during storm events. The concentrations of DIN in Tokyo were significantly higher than those from the other regions (*P* < 0.01), with an average of 142 *μ*mol L^−1^, followed by Nagano (at 37 *μ*mol L^−1^), Kochi (at 21 *μ*mol L^−1^), Mie (at 11 *μ*mol L^−1^), and Aichi (at 10 *μ*mol L^−1^). The spatial patterns of DIN during storm events were consistent with our previous study during baseflow conditions [2]. The regional pattern of DTN was similar to DIN, but the highest concentration of DON existed in Mie and with a completely different regional pattern to that during baseflow conditions: Mie (44 *μ*mol L^−1^) > Nagano (13 *μ*mol L^−1^) > Aichi (7 *μ*mol L^−1^) > Tokyo (6 *μ*mol L^−1^) > Kochi (3.5 *μ*mol L^−1^), implying that there were different controlling factors for DIN and DON in forested ecosystems, especially during storm events [27].

The concentrations of all the forms of P in the storm runoff in Nagano were the highest amongst the five regions with averages of 0.77, 0.37, and 0.40 *μ*mol L^−1^ for DTP, DIP, and DOP, respectively (Table 3). Additionally, a consistent regional pattern was found for the concentration of DTP, DIP, and DOP in storm runoff, being Nagano > Tokyo > Kochi > Mie > Aichi. However, there was an inverse order between Nagano and Tokyo when compared with the regional pattern of DIN during both storm and baseflow conditions. The dissolved inorganic phosphorus and total dissolved P in Nagano and Tokyo were significantly (*P* < 0.01) greater than those in other three regions. Although there was great diversity in climate, forest type, and geological conditions among the regions, taking account into the similar spatial patterns of the different dissolved P constituents (Table 3) and the significantly positive correlation between them (Table 2), we were convinced that the same potential factors controlling the dissolved P runoff during storm events were operating on a large spatial scale rather than just in each watershed.

As for the N : P ratio in the five regions, a distinct regional pattern was shown, with significantly lower N : P ratios in Kochi than those in the four other sites (*P* < 0.05; Table 3). Interestingly, different spatial patterns relating to the N : P ratio of storm flow (Kochi < Nagano < Mie < Aichi < Tokyo) were found when comparing them with baseflow conditions (Kochi < Mie < Aichi < Tokyo < Nagano) [2]. These results indicated that the “N-saturation” region, that is, Tokyo, would be more affected by being P-limited than the other four regions during storm events when N inputs from atmospheric sources increased.

### 3.3. Different Responses to Nutrient Concentrations and the N : P Ratio in Evergreen Conifer and Deciduous Broadleaf Forests

**Figure 1:**
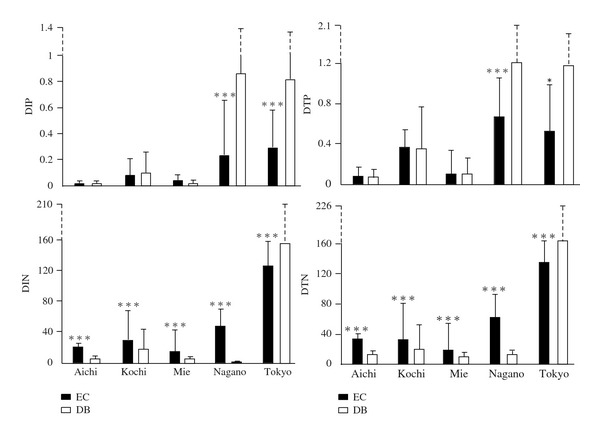
Forested stream nutrient concentrations for the different vegetation types in the five regions during storm events. The symbol on the top of columns shows a significant difference at either ***P ≤ 0.001, **0.001 ≤ *P* ≤ 0.01, or *0.01 ≤ *P* ≤ 0.05.

We found no significant differences in the DIP concentration between the DB and EC in the four regions including Aichi, Kochi, and Mie while significantly higher DIP was shown in the DB than that of the EC in Nagano and Tokyo (*P* < 0.001; top and left in Figure 1). Concentrations of DTP in the DB were significantly greater than those of the EC in Nagano (*P* < 0.001) and Tokyo (*P* < 0.001; top and right in Figure 1). However, the differences in N concentrations between the two forests were more significant when comparing the differing P constituents. Concentrations of DIN (mainly in the form of NO_3_–N) in the EC were significantly higher than those in the DB for Aichi (*P* < 0.001), Kochi (*P* < 0.001), Mie (*P* < 0.001), and Nagano (*P* < 0.001) while there was an inverse trend for Tokyo (*P* < 0.001  ; bottom and left in Figure 1). The similar site responses of DTN in storm flow to DIN are shown in each region (bottom and right in Figure 1). The different responses of the two types of vegetation to the differing nutrient concentrations as a result of storm flow was a consistently higher N : P ratio (DTN : DTP) in the EC than in the DB in each region (right part of Figure 1), which is also in accordance with those during baseflow conditions [2]. Therefore, it could be implied that the water bodies downstream of the EC will be more affected by being P-limited than the DB streams considering the increasing amounts of N inputs from atmospheric sources [24, 29] and the natural low fertility of the forest soil [30]. 

## 4. Discussion

### 4.1. Factors Controlling the Spatial Pattern of Nutrient Concentrations and the N : P Ratio in Storm Runoff

To compare the relative impacts of the two main variables, that is, region and vegetation (DB, EC), we calculated the weighting of the two factors with respect to the nutrient concentration and the N : P ratio using the whole dataset from the five regions. All *F* values for the regional factors were higher than those for the differing vegetation types, suggesting that region plays a more important role in controlling the nutrient concentrations and the N : P ratio under storm conditions.

### 4.2. Potential Limiting Nutrients in Storm Runoff Waters from Forested Watersheds

**Figure 2:**
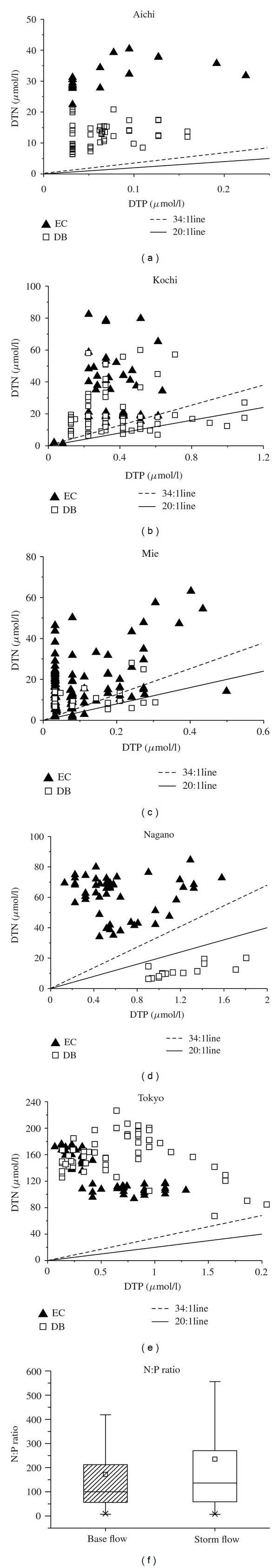
Relationships between the runoff concentrations of DTN and DTP in the EC and DB forests during storm events in each region.

To quantitatively describe the limiting nutrients, we assumed that phototrophic bacteria are likely to be limited by N when the ambient N : P ratio is less than 20, and limited by P when the N : P ratio is greater than 34 [25, 31]. Trends in DTP and DTN concentration by vegetation type for each region are shown in Figure 2. Three patterns could generally be determined according the size of the N : P ratios. Values located above the 34 : 1 line indicate P-limited conditions; between two lines for nonnutrient limited and on the 20 : 1 line as being N-limited. We found almost all the N : P ratios were greater than 34, except the values for the DB in Nagano, 26% and 4% of the values for the DB and EC, respectively, in Kochi, 11% and 4% of the values for the DB and EC in Mie, the ratios being mostly located near the 34 : 1 line. From the plots in Aichi and Tokyo, it could be concluded that storm water was generally P-limited from both the DB and EC forested streams. However, in Nagano, all storm water from the EC-forested streams was P-limited while storm water from the DB streams was N-limited. In Kochi, 96 and 74% of the storm water from EC and DB streams in storm events would be categorized as being P-limited while 4 and 26% of the storm water from the EC and DB forested streams in storm events would be N-limited. In Mie, for the EC and DB about 96 and 89% of the storm water were P-limited, respectively, and likewise 4 and 11% of the storm water from the EC and DB forested streams were N-limited. Generally, the N : P ratios of the EC sites were located to the left of the 34 : 1 line than those for the DB forests. These results suggest that the storm water from the EC streams should be more P-limited than that from the DB-streams, which is consistent with our previous study [2]. Moreover, the N : P ratios in the storm water were higher than under baseflow condition (bottom and right in Figure 2), implying that the storm water should be more sensitive to being P-limited relative to baseflow conditions.

## 5. Conclusions

In this study, the concentrations of dissolved N and P in storm water from forested watersheds in five regions in Japan during storm events were analyzed and compared. First, the concentrations of DIN, DON, DOP, DTN, DTP, and the N : P ratio were higher during storm events relative to baseflow conditions at all the sites, and the same spatial pattern for DIN in storm water was found as for baseflow conditions. Additionally, the spatial patterns across the five regions for all P constituents including DIP, DOP, and DTP followed the same order as being the greatest in Nagano > Tokyo > Kochi > Mie > Aichi, a little different from that of the trends in DIN during both storm and baseflow conditions, in which there was an inverse order for Nagano and Tokyo. The differences in concentrations and spatial patterns of nutrients between stormflow and baseflow conditions across the five regions verified the different mechanisms controlling the nutrient runoff during these two stream states.

Second, significantly higher concentrations of DIN were found in storm water from the EC sites than from the DB with the exception of Tokyo. However, the DTP concentrations from the EC were significantly lower than those from the DB in Nagano and Tokyo, with no significant differences evident between the two types of vegetation in the other three regions. Interestingly, a consistently higher N : P ratio in the storm water from the EC than those of the DB among each region were indicated, which is also in accordance with those measured during baseflow conditions.

Finally, almost all the N : P ratios in the storm water were generally higher than 34 with the exception of those from the DB in Nagano and a very small fraction of the sites in Kochi and Mie, implying that forested stream water in storm events is P-limited, especially in Tokyo, and at the EC sites in Nagano. When comparing the N : P ratios during baseflow conditions, there should be a greater sensitivity to P limitation in the storm water from forested watersheds to increasing trends in atmospheric N deposition and extreme storm events, more of which are possible in the future.

## Supplementary Material

We chose a set of headwater streams in the watersheds (Figure 1) across five regions (Aichi, 12 Kochi, Mie, Nagano, and Tokyo) In each region we selected four to seven streams with similar geological environments, where the main vegetation, including evergreen conifer (EC) plantation, natural deciduous broadleaf (DB), is representative of the region. The general information for the five regions and the watersheds, including the watershed areas, proportions of EC and DB, etc. is presented in Table 1, and more information is available from Zhang (2007).

## Figures and Tables

**Table 1:** Summary of forested runoff nutrient concentrations (*μ*mol L^−1^) and the molar N : P ratio in storm and baseflow states for the complete dataset (all sites).

Condition	Value	DTP	DTN	DIP	DIN	DOP	DON	N : P Ratio
Storm	Min	0.02	1.36	0.02	0.18	0.02	0.43	7
	Max	2.29	226.36	2.26	210.72	1.45	416	2567
	Mean	0.33	51.62	0.12	45.95	0.21	17.23	236
	SD	0.34	56.32	0.21	55.91	0.21	32.53	279

Baseflow*	Min	0.03	0.75	0.02	0.60	0.02	0.18	7
	Max	1.23	112	1.03	106	0.41	14.1	1858
	Mean	0.16	24.6	0.13	21.5	0.09	3.59	165
	SD	0.21	25.2	0.17	23.6	0.07	2.52	198

N : P ratios are defined as DTN : DTP molar ratios; molar N: *P* ≈ 0.45 N : P (by mass); *data from Zhang et al. [2].

**Table 2:** Relationships between the different forms of N and P.

*P*						
*r*	DTP	DTN	DIP	DIN	DOP	DON
DTP		<0.001	<0.001	<0.001	<0.001	<0.001
DTN	0.466**		<0.001	<0.001	<0.001	<0.001
DIP	0.798**	0.374**		<0.001	<0.001	<0.001
DIN	0.427**	0.992**	0.360**		0.062	<0.001
DOP	0.804**	0.371**	0.284**	0.321**		<0.001
DON	0.136**	0.151**	0.135**	0.190**	0.081	

**Correlation is significant at the *P* < 0.01 level.

**Table 3:** Means for nutrient concentrations (*μ*mol L^−1^) and the molar N : P ratio at the 20 sites across the five study regions in Japan (standard deviations are in parentheses).

Site	Number of samples	DTP	DTN	DIP	DIN	DOP	DON	N : P ratio
EC-A3	19	0.07 (0.06)	31.73 (4.71)	0.02 (0.01)	21.38 (7.56)	0.06 (0.05)	10.35 (10.11)	638 (296.37)
DB-A4	53	0.06 (0.04)	11.95 (3.92)	0.02 (0.01)	6.16 (3.57)	0.04 (0.03)	5.79 (1.44)	241 (129.75)
**Aichi**	**72**	**0.06** **(0.04) **	**17.17** **(9.69) **	**0.02** **(0.01) **	**10.18** **(8.33) **	**0.05** **(0.04) **	**7.00** **(5.62) **	**346** **(256.25) **

DB-K2	24	0.48 (0.29)	14.86 (3.22)	0.14 (0.16)	12.75 (2.77)	0.34 (0.14)	2.11 (0.94)	40 (23.19)
DB-K3	64	0.30 (0.16)	20.90 (14.27)	0.09 (0.12)	17.24 (12.48)	0.22 (0.12)	3.66 (2.88)	79 (47.65)
EC-K4	4	0.06 (0.03)	1.74 (0.27)	0.20 (0.00)	0.26 (0.16)	0.04 (0.23)	1.47 (0.30)	40 (23.36)
EC-K5	5	0.41 (0.16)	37.67 (2.86)	0.04 (0.05)	34.58 (2.53)	0.37 (0.18)	3.09 (0.79)	107 (48.05)
EC-K6	21	0.42 (0.11)	18.94 (1.65)	0.05 (0.05)	16.56 (1.51)	0.37 (0.12)	2.38 (0.69)	49 (15.97)
EC-K7	19	0.35 (0.11)	54.53 (15.14)	0.16 (0.99)	47.90 (11.98)	0.19 (0.15)	6.62 (5.04)	169 (70.19)
**Kochi**	**137**	**0.35** **(0.19) **	**24.26** **(17.37) **	**0.10** **(0.12) **	**20.74** **(15.34) **	**0.26** **(0.15) **	**3.52** **(3.08) **	**80** **(59.28)**

EC-M1	29	0.06 (0.08)	15.17 (11.63)	0.02 (0.00)	12.01 (8.47)	0.05 (0.08)	53.97 (50.00)	352 (221.19)
EC-M2	40	0.13 (0.10)	13.87 (11.89)	0.04 (0.02)	10.01 (9.86)	0.10 (0.10)	53.77 (39.13)	117 (76.00)
EC-M3	32	0.08 (0.09)	16.92 (8.67)	0.03 (0.04)	13.18 (7.43)	0.06 (0.08)	52.36 (31.13)	370 (248.20)
EC-M4	4	0.10 (0.08)	20.56 (10.33)	0.02 (0.00)	9.31 (0.74)	0.08 (0.07)	157.4 (150.69)	433 (471.02)
EC-M5	10	0.12 (0.13)	37.93 (12.19)	0.02 (0.00)	34.46 (11.77)	0.11 (0.13)	48.70 (26.98)	670 (493.82)
DB-M8	39	0.09 (0.09)	9.46 (4.75)	0.03 (0.18)	5.44 (2.69)	0.07 (0.08)	4.01 (3.61)	182 (107.83)
**Mie**	**154**	**0.10** **(0.10) **	**15.40** **(11.68)**	**0.03** **(0.02)**	**11.44** **(10.18)**	**0.07** **(0.09)**	**43.95** **(50.03)**	**275** **(261.32)**

EC-N2	24	0.56 (0.50)	68.36 (6.18)	0.14 (0.46)	61.91 (8.01)	0.42 (0.31)	6.45 (4.79)	190 (112.23)
EC-N4	9	1.01 (0.22)	53.34 (14.74)	0.24 (0.07)	5.05 (2.86)	0.78 (0.17)	48.29 (12.11)	52.67 (7.04)
EC-N5	20	0.61 (0.24)	56.75 (13.60)	0.34 (0.06)	50.20 (13.68)	0.27 (0.25)	6.55 (2.13)	100 (32.62)
DB-N6	15	1.20 (0.29)	11.50 (4.32)	0.87 (0.20)	0.77 (0.83)	0.33 (0.10)	10.73 (3.66)	9 (2.67)
**Nagano**	**68**	**0.77** **(0.44) **	**50.41** **(23.73) **	**0.37** **(0.40) **	**37.45** **(27.95) **	**0.40** **(0.29) **	**12.96** **(15.02) **	**106** **(98.13)**

EC-T5	57	0.51 (0.33)	133.92 (28.21)	0.30 (0.32)	126.93 (30.28)	0.22 (0.16)	6.98 (4.83)	483 (464.92)
DB-T6	60	0.70 (0.46)	161.54 (31.80)	0.29 (0.27)	156.38 (32.97)	0.41 (0.25)	5.16 (5.38)	390 (297.56)
**Tokyo**	**117**	**0.61** **(0.41)**	**148.08** **(33.02)**	**0.29** **(0.30)**	**142.03** **(34.8)**	**0.32** **(0.23) **	**6.05** **(5.18)**	**435** **(389.30) **

## References

[1] Dana RW, Emily SB, Robert OHJ, Gene EL (2007). Forest age, wood and nutrient dynamics in headwater streams of the Hubbard Brook Experimental Forest, NH. *Earth Surface Processes and Landforms*.

[2] Zhang Z, Fukushima T, Shi P (2008). Baseflow concentrations of nitrogen and phosphorus in forested headwaters in Japan. *Science of the Total Environment*.

[3] Jobbágy EG, Jackson RB (2001). The distribution of soil nutrients with depth: global patterns and the imprint of plants. *Biogeochemistry*.

[4] Reich PB, Oleksyn J (2004). Global patterns of plant leaf N and P in relation to temperature and latitude. *Proceedings of the National Academy of Sciences of the United States of America*.

[5] Raison RJ, Partap KK (2011). Possible impacts of climate change on forest soil health. *Soil Health and Climate Change*.

[6] Vitousek PM, Howarth RW (1991). Nitrogen limitation on land and in the sea: how can it occur?. *Biogeochemistry*.

[7] Klausmeler CA, Litchman E, Daufreshna T, Levin SA (2004). Optimal nitrogen-to-phosphorus stoichiometry of phytoplankton. *Nature*.

[8] Elser JJ, Bracken MES, Cleland EE (2007). Global analysis of nitrogen and phosphorus limitation of primary producers in freshwater, marine and terrestrial ecosystems. *Ecology Letters*.

[9] Elser JJ, Elser MM, Mackay NA, Carpenter SR (1988). Zooplankton-mediated transitions between N- and P-limited algal growth. *Limnology & Oceanography*.

[10] Townsend AR, Cleveland CC, Asner GP, Bustamante MMC (2007). Controls over foliar N:P ratios in tropical rain forests. *Ecology*.

[11] Robert LS, Christian LL, Michael NW (2008). Stoichiometry of soil enzyme activity at global scale. *Ecology Letters*.

[12] Yuan ZY, Chen HYH (2009). Global-scale patterns of nutrient resorption associated with latitude, temperature and precipitation. *Global Ecology and Biogeography*.

[13] Yuan ZY, Chen HYH (2009). Global trends in senesced-leaf nitrogen and phosphorus. *Global Ecology and Biogeography*.

[14] Jason CN, Sarah EH, Peter MV (2000). Nutrient and mineralogical control on dissolved organic C, N and P fluxes and stoichiometry in Hawaiian soils. *Biogeochemistry*.

[15] Stefand M, John AT, Robert BJ, Amilcare P (2010). Stoichiometric controls on carbon, nitrogen, and phosphorus dynamics in decomposing litter. *Ecological Monographs*.

[16] Jackson RB, Mooney HA, Schulze ED (1997). A global budget for fine root biomass, surface area, and nutrient contents. *Proceedings of the National Academy of Sciences of the United States of America*.

[17] Megan EM, Tanguy D, Lars OH (2004). Scaling of C:N:P stoichiometry in forests worldwide: implications of terrestrial redfield-type ratios. *Ecology*.

[18] Christopher PC, Jeffrey JM (1997). Linking the hydrologic and biogeochemical controls of nitrogen transport in near-stream zones of temperate-forested catchments: a review. *Journal of Hydrology*.

[19] Myron JM (2001). Linkages of nitrate losses in watersheds to hydrological processes. *Hydrological Processes*.

[20] Hideaki S, Osamu S, Hisano T (2004). Nitrogen dynamics in the hyporheic zone of a forested stream during a small storm, Hokkaido, Japan. *Biogeochemistry*.

[21] John DS, Javier FE, Christopher AK, Megan EM, Steven AT, Zhang LX (2005). A conceptual framework for ecosystem stoichiometry: balancing resource supply and demand. *Oikos*.

[22] Mulholland PJ (1992). Regulation of nutrient concentrations in a temperate forest stream: roles of upland, riparian, and instream processes. *Limnology &amp; Oceanography*.

[23] Brown VA, McDonnell JJ, Burns DA, Kendall C (1999). The role of event water, a rapid shallow flow component, and catchment size in summer stormflow. *Journal of Hydrology*.

[24] Roderick CD, Oskar F, Annikki M, Ross EM, Harry TV (2009). Optimal function explains forest responses to global change. *BioScience*.

[25] Turner BL, Baxter R, Whitton BA (2003). Nitrogen and phosphorus in soil solutions and drainage streams in Upper Teesdale, northern England: implications of organic compounds for biological nutrient limitation. *Science of the Total Environment*.

[26] Ohte N, Tokuchi N, Katsuyama M, Hobara S, Asano Y, Koba K (2003). Episodic increases in nitrate concentrations in streamwater due to the partial dieback of a pine forest in Japan: runoff generation processes control seasonality. *Hydrological Processes*.

[27] Zhang Z, Fukushima T, Onda Y (2007). Nutrient runoff from forested watersheds in central Japan during typhoon storms: implications for understanding runoff mechanisms during storm events. *Hydrological Processes*.

[28] Paruelo JM, Burke IC, Lauenroth WK (2001). Land-use impact on ecosystem functioning in eastern Colorado, USA. *Global Change Biology*.

[29] Steven SP, Lars OH (2002). Nitrogen loss from unpolluted South American forests mainly via dissolved organic compounds. *Nature*.

[30] Bobbink R, Hornung M, Roelofs JGM (1998). The effects of air-borne nitrogen pollutants on species diversity in natural and semi-natural European vegetation. *Journal of Ecology*.

[31] Sakamoto M (1966). Primary production by phytoplankton community in some Japanese lakes and its dependence on lake depth. *Archiv für Hydrobiologie*.

