# Supplementary figures and images for: N : P Stoichiometry in a Forested Runoff during Storm Events: Comparisons with Regions and Vegetation Types
Source: ScientificWorldJournal. 2012 Apr 1;2012:257392. doi: 10.1100/2012/257392 (PMC3322624; doi:10.1100/2012/257392)

Supplementary Fig. 1

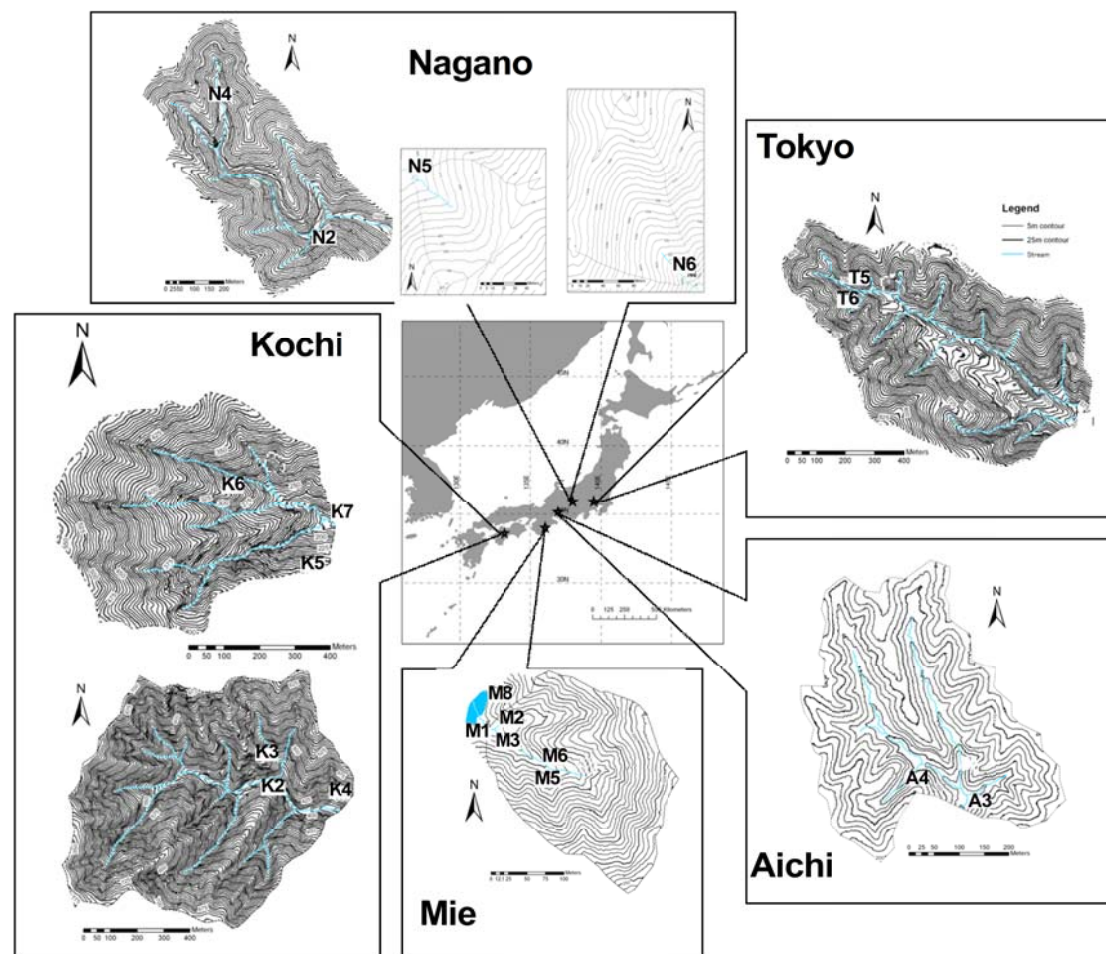

Supplement: Supplementary file 1 — We chose a set of headwater streams in the watersheds (Figure 1) across five regions (Aichi, 12 Kochi, Mie, Nagano, and Tokyo) In each region we selected four to seven streams with similar geological environments, where the main vegetation, including evergreen conifer (EC) plantation, natural deciduous broadleaf (DB), is representative of the region. The general information for the five regions and the watersheds, including the watershed areas, proportions of EC and DB, etc. is presented in Table 1, and more information is available from Zhang (2007). [file 257392.f1.pdf]
