# Supplementary material for: N : P Stoichiometry in a Forested Runoff during Storm Events: Comparisons with Regions and Vegetation Types
Source: ScientificWorldJournal. 2012 Apr 1;2012:257392. doi: 10.1100/2012/257392 (PMC3322624; doi:10.1100/2012/257392)
Supplement: Supplementary file 2 [file 257392.f2.pdf]

Supplementary Table 1

Summary information for each sampling site in the five study regions

Region	Location	Mean precipitation (mm yr^{-1})	Mean temperature (°C)	Watershed code	Watershed characteristic	M.E. (m)	Watershed area (ha)
Aichi	136º57.9´E 35º10.0´N	2108	15.4	A3	Cypress forested	161	3
				A4	DB forested	175	3.5
Kochi	133º07.7´E 33º12.4´N	2782	14.6	K2	DB forested	381	45.3
				K3	DB forested	556	4.9
				K4	Cedar forested	481	2.0
				K5	Cedar and cypress	470	55.7
				K6	Cypress forested	381	5.7
				K7	Cypress forested	368	33.2
Mie	136º23.4´E 34º26.9´N	2560	14.2	M1	Cypress forested	180	4.9
				M2	Cypress forested	161	1.2
				M3	Cypress forested	183	3.5
				M4	Cypress forested	152	0.1
				M5	Cypress forested	181	0.3
				M8	DB forested	133	0.2
Nagano	138º22.5´E 36º52.4´N	1487	10.8	N2	Cypress forested	1153	25
				N4	Cypress forested	1205	4.7
				N5	Larch forested	1153	25
				N6	DB forested	1270	1.8
Tokyo	139º18.7´E 35º47.3´N	1504	13.6	T5	Cedar and Cypress	770	1.3
				T6	DB forested	778	1.6

Footnotes:

-Watershed codes are showed in Fig.1;

- M.E. in the table means the medium elevation;

- DB is the deciduous broadleaf type of forest;

-Cypress: *Chamaecyparis obtuse*; Cedar: *Cryptomeria japonica*.
